# Tarsal morphology of ischyromyid rodents from the middle Eocene of China gives an insight into the group’s diversity in Central Asia

**DOI:** 10.1038/s41598-021-90796-1

**Published:** 2021-06-02

**Authors:** Łucja Fostowicz-Frelik, Sergi López-Torres, Qian Li

**Affiliations:** 1 Key Laboratory of Vertebrate Evolution and Human Origins, Institute of Vertebrate Paleontology and Paleoanthropology, Chinese Academy of Sciences, Beijing, 100044 China; 2 CAS Center for Excellence in Life and Paleoenvironment, Beijing, China; 3 Institute of Paleobiology, Polish Academy of Sciences, 00-818 Warsaw, Poland; 4 Division of Paleontology, American Museum of Natural History, New York, NY 10024 USA; 5 New York Consortium in Evolutionary Primatology, New York, NY USA

**Keywords:** Evolution, Zoology, Ecology

## Abstract

Ischyromyids are a group of large rodents with the earliest fossil record known from the late Paleocene (Clarkforkian) of North America; they are considered the earliest fossil representatives of Rodentia of modern aspect. Ischyromyids dominated early Paleogene small-mammal assemblages of North America and in the latest Paleocene migrated to western Europe and to Asia; in the latter they survived only to the beginning of the late Eocene, but were never abundant. Here we describe for the first time the calcanei of ischyromyids from the early middle Eocene of the Erlian Basin in Nei Mongol, northern China. These calcanei document the existence of three species. The morphology of the studied tarsal bones overall suggests ambulatory locomotion for these animals (‘slow cursors’), similar to that of the coypu and porcupines, but one form shows more marked cursorial capabilities. These differences show that Chinese ischyromyids, although rare, had attained greater taxonomic diversity by the middle Eocene in Nei Mongol than estimated from dental remains. We also address the question of the morphological and ecological divergence of these ischyromyids in relation to their North American counterparts, as well as the issue of a direct dispersal route from North America to Asia in the early Eocene.

## Introduction

Ischyromyidae are the group that includes the earliest and most basal rodents, sometimes regarded as a stem rodent group^1,2^ or grouped with ctenodactyloids (*Cocomys*) as representing the first true rodents and a sister clade to Alagomyidae^3^; the latter were removed from rodents to Rodentiaformes by Meng and Wyss^2^. Whether ischyromyids are more derived than basal ctenodactyloids has been a matter of dispute^4–8^. Nevertheless, the appearance of ischyromyids predates that of the earliest ctenodactyloids by ca. one million years, which still constitutes the earliest fossil record of rodents^3,6,9,10^.

As currently presumed, ischyromyids originated in North America. Their earliest fossil record consists of *Acritoparamys atavus* (Bear Creek, Montana, USA) and *Paramys adamus* (Big Multi Quarry, Wyoming, USA), both dated at the latest Paleocene, early Clarkforkian North American Land Mammal Age (NALMA; see^6,11^). The group rapidly became diversified and thrived in North America during the Eocene^6,12^. Ischyromyids quickly migrated to Europe, where they are known at least from the beginning of the Ypresian (earliest Eocene; Dormaal [MP 7] faunal level; see^13,14^) and possibly even from the latest Paleocene (MP 6b faunal level^15^). However, they were much less diverse and abundant in Europe than in North America. Ischyromyids appeared in Asia in the earliest Eocene^16^; thus, the Asian fossil record of this group^17–19^ is only marginally younger than the European one. Also, they were never species-rich or common faunal elements on this continent^17,19–22^. Recently, Mein and Pickford^23^ reported the first record of an ischyromyid from the middle Eocene of Namibia. If the family attribution of *Namaparamys* is correct, Africa would be the final continent colonized by this group.

**Figure 1:**
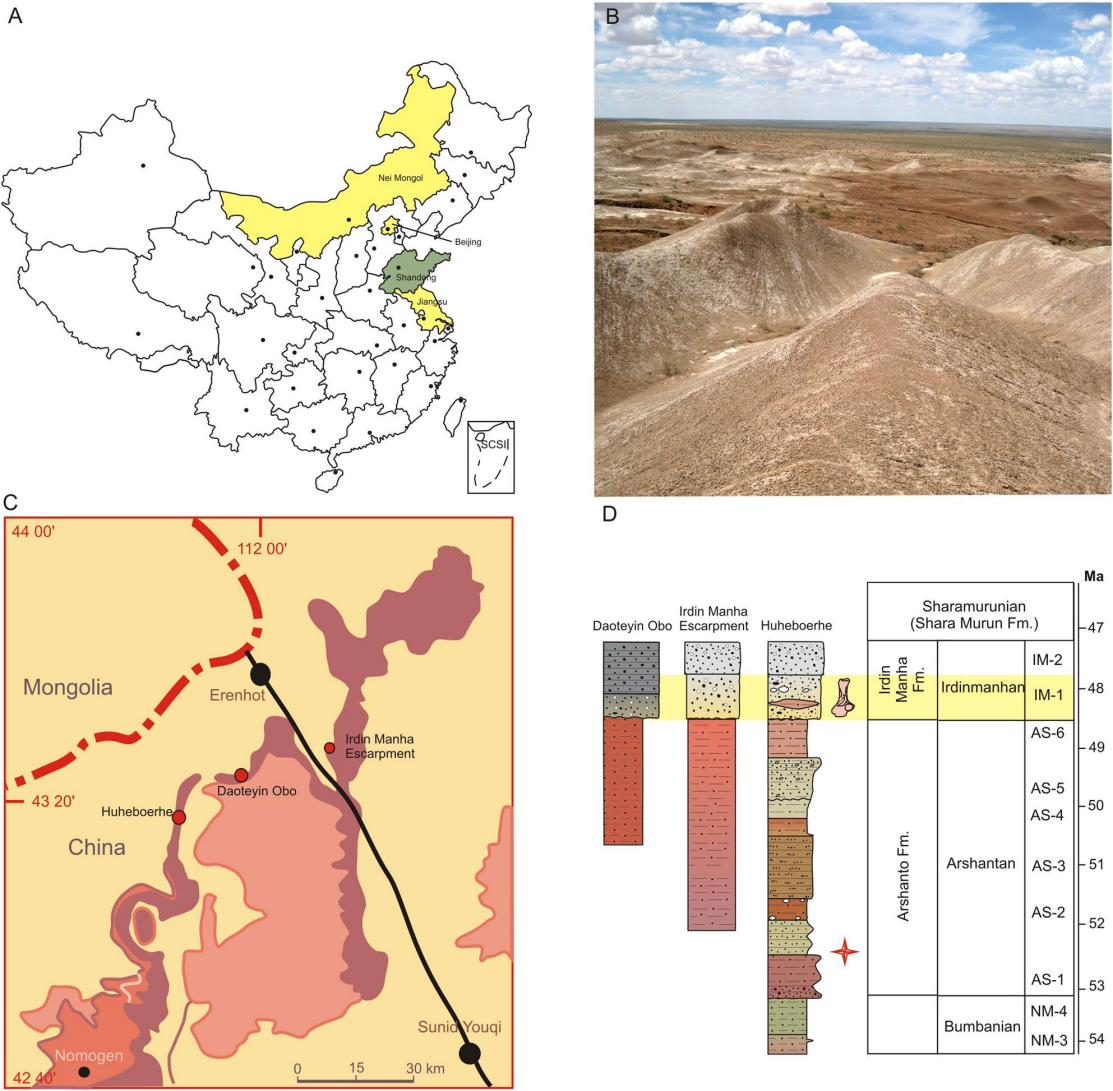
Stratigraphic and geographic distribution of Ischyromyidae in China. (**A**) map of ischyromyid findings in China; yellow denotes Irdinmanhan findings in a province, green denotes older (?Bumbanian) findings; (**B**) panoramic view of Huheboerhe area; (**C**) detailed map of studied sites in the Erlian Basin; (**D**) stratigraphy of studied localities in the Erlian Basin, Nei Mongol, China; Irdinmanhan strata marked in yellow. (Maps and stratigraphic section created in Corel Draw X4 (v.14.0.0.567) by Łucja Fostowicz-Frelik; photograph taken by Łucja Fostowicz-Frelik).

Here we present the postcranial material of ischyromyids from Asia for the first time. Our findings come from the middle Eocene localities in the Erlian Basin (Fig. 1), which is one of the classic Paleogene fossiliferous areas in Nei Mongol, northern China^10,19,24–28^. Despite the overall diversity and abundance of rodents during the Eocene in Nei Mongol^29^, ischyromyids were always very rare there (except for *Asiomys*;^19,29^). Our study of the calcanei of ischyromyids from the Erlian Basin shows two or three different taxa (either genera or species). This diversity points to a greater species richness of this group in northern China during the middle Eocene than was previously suggested by the dental material^19,21^, comparable to the record from southern China (Shanghuang;^30^). Furthermore, differences in the calcaneal structure between Asian and North American taxa imply a different paleoecology for Asian taxa with respect to their North American predecessors and a different composition of Eocene rodent paleocommunities on both continents.

*Institutional abbreviations* AMNH, American Museum of Natural History, New York, NY, USA; ISEZ, Institute of Systematics and Evolution of Animals, Polish Academy of Sciences, Cracow, Poland; IVPP, Institute of Vertebrate Paleontology and Paleoanthropology, Chinese Academy of Sciences, Beijing, China; ZMCAS, Zoological Museum of the Chinese Academy of Sciences, Beijing, China.

## Results

### Systematic paleontology

Order Rodentia Bowdich, 1821^31^

Family Ischyromyidae Alston, 1876^32^

Genus *Asiomys* Qi, 1987^33^

*Asiomys dawsoni* Qi, 1987^33^

Figure 3A–E

*Material*. Fragment of right calcaneus (IVPP V24417), early Middle Eocene, Huheboerhe, Irdin Manha Formation, Erlian Basin, China.

*Description*. The bone is damaged and most probably that of a juvenile as it shows loss of the tissue in the extremities of the bone such as the calcaneal tuber and calcaneal eminence, which are usually less calcified in juveniles. The bone is relatively large (Table 1), with an elongated calcaneal tuber and a relatively short body (Fig. 3A–D). The sustentaculum tali is partly damaged; it has a subcircular articulation facet, which was probably more extended craniocaudally than mediolaterally. The caudal margin of the sustentaculum tali is inclined cranially, similar to the condition seen in species A and more than in species B (Fig. 3A). The sustentacular facet overlaps about one-half of the craniocaudal reach of the ectal facet. The groove for the ‘spring ligament’ (sensu Szalay and Decker^34^), which runs along the medial edge of the sustentaculum tali, is poorly pronounced. Likewise, the calcaneal groove for the tendon of the flexor fibularis muscle is shallow and poorly marked, most probably due to poor preservation. The ectal facet is relatively wide and similarly shaped as in species B (below). The peroneal process is completely damaged.

**Table 1:** Measurements (in mm) of ischyromyid calcanei from the early middle Eocene of the Erlian Basin, Nei Mongol, China.

Specimen/measurement	CL	CW	BL	TW	TT	EL	AEW	TEW	CMT	BW	CCW	CCL	TWM	TL
*Asiomys dawsoni* IVPP V24417	–	11.0	7.5	–	–	6.8	3.5	5.7	9.8	–	5.9	5.6	4.9	–
Species A IVPP V24416	26.0	15.0	7.5	6.8	7.7	8.4	5.5	6.5	11.0	10.3	7.8	6.0	4.8	15.4
Species B IVPP V24418	28.6	14.3	8.0	7.45	9.6	9.8	4.4	6.0	11.5	10.5	8.8	6.7	5.5	16.5

**Figure 2:**
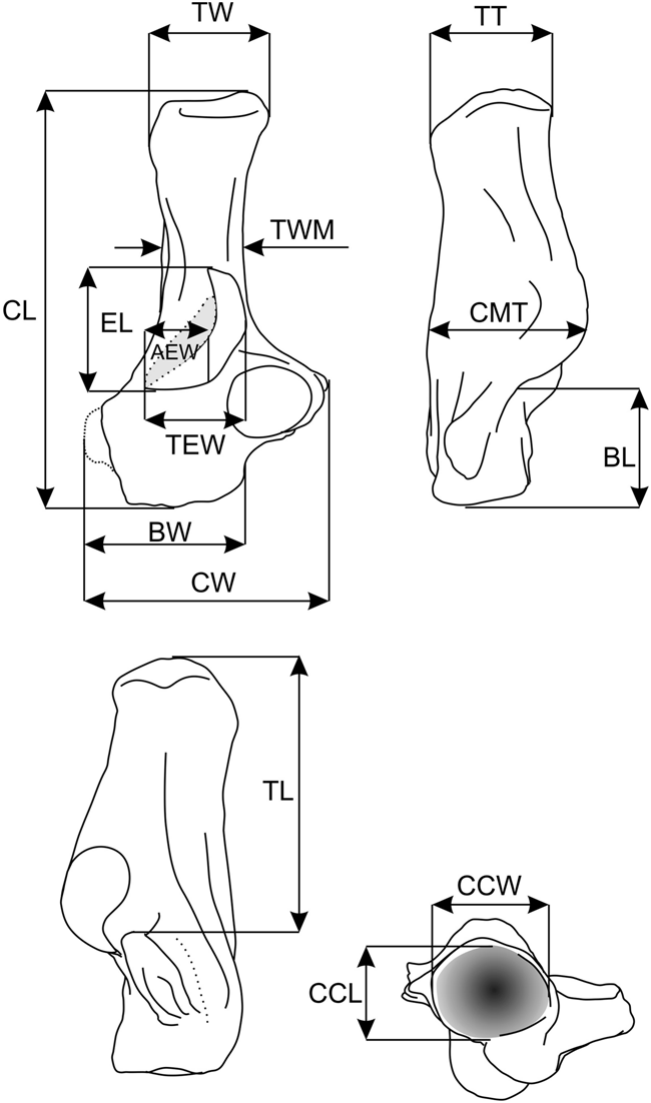
Linear measurements of the calcaneus. Abbreviations: AEW, ectal facet anterior width; BL, calcaneal body length; BW, calcaneal body width; CCL, calcaneocuboid facet length; CCW, calcaneocuboid facet width; CL, calcaneus length; CMT, calcaneus maximum thickness; CW, calcaneal width; EL, ectal facet length; TEW, ectal facet total width; TL, tuber calcanei length; TT, tuber calcanei thickness; TW, tuber calcanei width; TWM, tuber calcanei width in mid-length. (Figure created in Corel Draw X4 (v.14.0.0.567) by Łucja Fostowicz-Frelik).

For measurements and abbreviation definitions, see Fig. 2.

**Figure 3:**
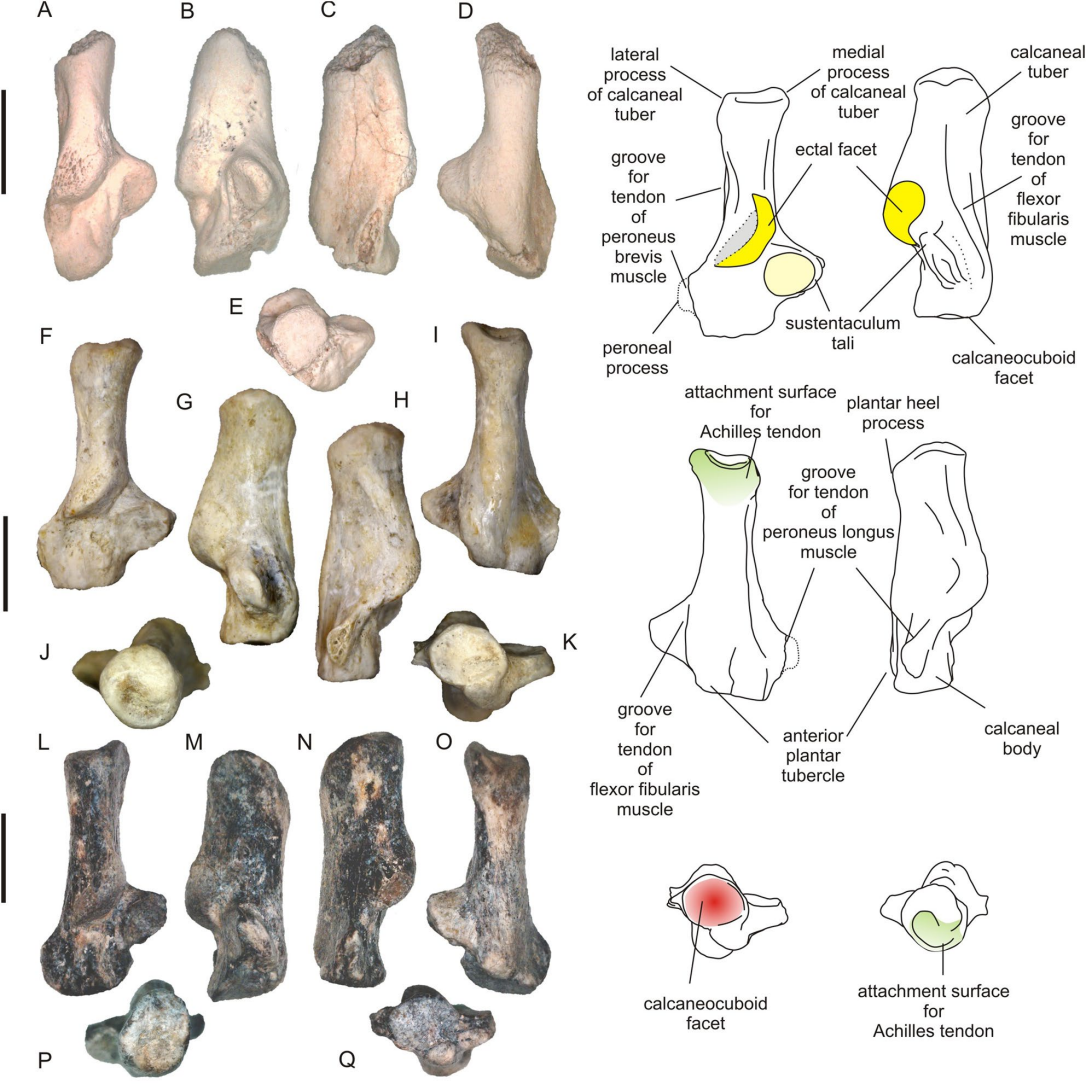
Ischyromyid calcanei from the early middle Eocene of the Erlian Basin, Nei Mongol, China. (**A**–**E**), *Asiomys dawsoni* (IVPP V24417), right calcaneus, juvenile?; (**F**–**K**), species A (IVPP V24416), right calcaneus, adult; (**L**–**Q**), species B (IVPP V24418), right calcaneus, adult. In: A, F and L, dorsal; B, G and M, medial; C, H and N, lateral; D, I and O plantar; J and P caudal; E, K and Q, cranial views. Explanatory line drawings (right side) show important morphological features. Note sustentacular facet marked pale yellow. Scale bar equals 10 mm. (Photographs taken by Łucja Fostowicz-Frelik; drawings created in Corel Draw X4 (v.14.0.0.567) by Łucja Fostowicz-Frelik).

The calcaneal tuber is strongly compressed, but it resembles in shape those of species A and B. A long groove for the calcaneofibular ligament is impressed on its lateral side.

The anterior plantar tubercle is large and swollen, similar to that in species A, and touches the brim of the calcaneocuboid surface. The latter, only slightly damaged laterally, is round in outline, without a distinct pit, and inclined about 20–30°.

Systematic remark: The fossil was associated with *Asiomys* dentition found in the same spot. We attribute specimen IVPP V24417 to *Asiomys dawsoni*, based on this fact and its distinctive size (*Asiomys* being the largest rodent in the assemblage). *Asiomys* is the only ischyromyid rodent known from the basal strata of the Irdin Manha Formation of Huheboerhe.

Genus indet.

Species A

Figure 3F–K

*Material.* Right calcaneus (IVPP V24416), early Middle Eocene, Irdin Manha Escarpment, Irdin Manha Formation, Erlian Basin, China.

*Description.* The right almost complete calcaneus of an adult specimen is relatively large (Table 1), comparable in length to the calcaneus of a coypu (*Myocastor coypus*) or Asiatic brush-tailed porcupine (*Atherurus macrourus*). The bone has a characteristically elongated calcaneal tuber and rather short body (Fig. 3F–I). The calcaneal tuber is quite slender in comparison with the structure found in the coypu and porcupines. The shape of the bone resembles most closely the calcaneus of *Paramys wortmani* (see^35^: Fig. 12B), although in *Paramys* the calcaneal tuber is more compressed mediolaterally.

The sustentaculum tali is large and eminent, reaching far medially and tapering, although its medial end forms a blunt edge parallel to the long axis of the bone. This medial edge also bears a well-marked but not deep groove of the calcaneonavicular (or ‘spring’) ligament (Fig. 3G). The sustentacular facet (facies articularis talaris media in Fostowicz-Frelik^36^: Fig. 12B2) is round, with only slight anteroposterior compression. It occupies almost the whole dorsal surface of the sustentaculum, encroaching slightly onto the calcaneal body. In that it differs from *Notoparamys* and *Paramys wortmani*, which both have a much more medially placed sustentacular facet, which does not encroach on the calcaneal body. The range of the sustentacular facet overlaps less than one-third of the ectal facet (posterior facies articularis talaris in Fostowicz-Frelik^36^: Fig. 12B2) on its anterior and medial sides. The calcaneal eminence is slightly longer than that in *Marmota* and *Sciurus*, in proportions closer to that of porcupines and of similar size as in *Paramys wortmani*. The ectal facet is wide, long, and has a distinctly helical course, even more strongly marked than in North American ischyromyids (see Rose and Chinnery^35^: Fig. 12A). It is, however, inclined more strongly mediolaterally than in *Notoparamys* and *Paramys*, and faces strongly medially. On the dorsal side of the calcaneal eminence, posterolateral to the ectal facet, there is a flattened rough area (finely pitted), marking the place of attachment of the lateral collateral ligaments binding the distal fibula and the astragalus with the calcaneus and stabilizing the astragalocalcaneal joint.

A calcaneal body is short and stocky with poorly marked tendon ridges at the dorsal surface. A large peroneal process is partly damaged at its lateral margin. The process is placed closer to the cuboid surface than the sustentaculum tali. The position of the sustentaculum tali and the proportions of the calcaneal body of specimen IVPP V24416 resemble rather closely the calcaneus of *Paramys wortmani* (see^35^).

The calcaneal tuber is not ‘pinched’ at its dorsal side but moderately compressed, thus there is no coracoid ridge posterior to the ectal facet. At the lateral side of the tuber, there is a long groove for the calcaneofibular ligament running askew, towards the dorsal surface of the calcaneal tuber. The groove for the calcaneofibular ligament is more weakly expressed than in the North American paramyines and arboreal sciurids, but similar to that of *Marmota*.

The caudal surface of the calcaneal tuber is subcircular (only slightly more extended dorsoplantarly than mediolaterally, see Fig. 3 and Table 1). The groove for the calcaneal tendon (= Achilles tendon) is deep and placed asymmetrically at the surface (Fig. 3J). Also, the medial process of the calcaneal tuber is much better developed and extending medially.

The plantar surface of the bone is almost straight with a delicate flexure cranially to a well-developed plantar heel process (Fig. 3G). The anterior plantar tubercle is relatively large, swollen, but shifted medially, towards the sustentaculum tali. It is placed very close to the cuboid surface, almost touching its margin; such location and the medial shifting resembles the condition in some ground squirrels, e.g., *Cynomys* (see Fostowicz-Frelik et al.^8^: Fig. 3D–F). The anterior plantar tubercle is also somewhat flattened and inclined medially and forms a well-marked calcaneal groove for the tendon of the flexor fibularis muscle.

The calcaneocuboid articular surface is semicircular, slightly wider mediolaterally than long dorsoplantarly, which distinguishes species A from *Marmota* and paramyines (see^35^). It is almost transversally positioned, not inclined, as in most of the rodent taxa (coypu and porcupines included), and gently concave; it is also confluent and level with the cuboid pit, forming one round surface at the cranial end of the bone.

Genus indet.

Species B

Figure 3L–Q

*Material* Right calcaneus (IVPP V24418), early Middle Eocene, Daoteyin Obo, Irdin Manha Formation, Erlian Basin, China.

*Description* The bone is complete, slightly larger than in species A (Table 1), matching in length the calcaneus of the coypu. Its overall structure is very similar to the calcaneus of *Paramys* (either *P*. *wortmani* or *P*. *taurus*, see Rose and Chinnery^35^: Fig. 12B, C). It has a long and strong calcaneal tuber and a relatively strong but short calcaneal body (Fig. 3L). The tuber is more compressed mediolaterally than in species A; thus, the caudal surface of the tuber is extended more dorsoplantarly than mediolaterally (Fig. 3P). The attachment for the calcaneal tendon forms a rounded concavity at the caudal side of the tuber, and is more horizontally and symmetrically located at the surface than in species A. The lateral surface of the calcaneal tuber bears a marked scar from the calcaneofibular ligament, although the scar is convex, not concave as in species A and in other compared taxa (e.g., *Cynomys*).

The sustentaculum tali is large and round; it is located relatively close to the calcaneal body, not extending as far medially as in the North American paramyines (see^35^). It is slightly longer anteroposteriorly and located more caudally (closer to the ectal facet) than in species A. Thus, the sustentacular surface overlaps ca. one-half of the cranial part of the ectal facet. The medial edge of the sustentacular shelf bears a deep groove for the ‘spring ligament’.

The ectal facet is large, equally wide throughout its length, long and helical, although its course is straighter along the proximodistal direction than in species A. The ectal surface faces mediodorsally, with a slightly weaker medial component than in species A. The dorsal surface of the tuber, just caudal to the ectal facet, is not typically ‘pinched’ into a sagittally oriented crest, but it is, nevertheless, more mediolaterally compressed than in the species A, similar to *Marmota*.

The calcaneal body forms about one-third of the bone length. Its dorsal surface is carved by deep longitudinal marks indicating the position of the extensor digitorum brevis muscle (Fig. 3). A middle-size peroneal process is located cranially at the calcaneal body. It is strong and long anteroposteriorly, reaching almost the edge of the calcaneocuboid surface. Its lateral edge shows a deep groove for the tendon of the peroneus longus muscle, while its dorsal surface forms a groove for the peroneus brevis muscle tendon (Fig. 3). Species B differs from the ground squirrels in the shape and location of the peroneal process, which is less extended laterally in species B than e.g., in marmots, although it is relatively much larger than in the coypu and porcupines.

The anterior plantar tubercle looks less swollen than in species A; it is located at the very margin of the calcaneocuboid surface and as in species A is shifted medially (Fig. 3O, Q). The calcaneocuboid surface is slightly inclined (ca. 25°) anteromedially, which distinguishes the bone from species A, *Marmota*, and *Notoparamys*, which all have the calcaneocuboid facet almost transversal and perpendicular to the long axis of the calcaneus. In this respect, the calcaneocuboid surface resembles more closely the calcaneus of *Paramys taurus* (Rose and Chinnery^35^: Fig. 12C). The calcaneocuboid surface is almost round, slightly wider mediolaterally, resembling that of species A. A relatively small calcaneal pit (extending only to a half of the anterior plantar tubercle base, see Fig. 3Q), smaller but deeper than in species A, forms a shallow sink at the medial side of the surface, cranially to the sustentaculum tali.

### PCA analysis

A Principal Component Analysis (PCA) was performed based on 14 measurements of the calcaneus. The analysis included the calcaneal measurements of five ischyromyid species (two described here as species A and B, and three comparative species from North America) and 16 extant large rodent species (Supplementary Table S1). The extant taxa represent six basic types of locomotor adaptations found in rodents: ambulatorial (terrestrial generalists), amphibious (swimming), arboreal (tree climbing), cursorial (four-pedal runners), ricochetal (bipedal jumpers), and semi-fossorial (burrowing).

**Figure 4:**
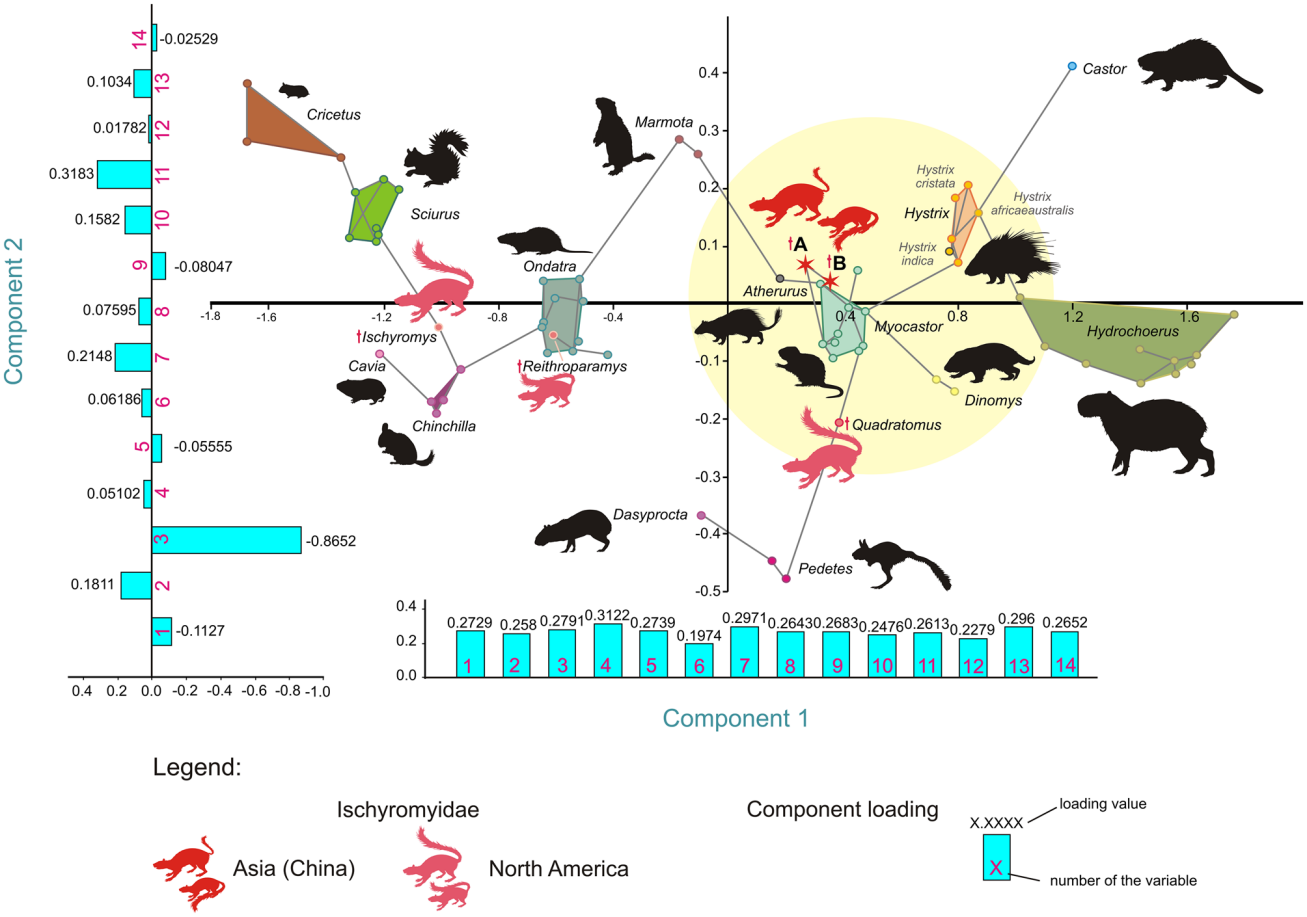
Principal component analysis of 14 metric parameters of rodent calcanei. The morphospace including paramyid calcanei from Nei Mongol in yellow circle. Lines connecting all data points represent a minimum spanning tree (MST) based on a Euclidean distance matrix. The loadings of the Components 1 and 2 shown at the corresponding axes. Strictly fossil taxa marked in red and pink, extant in black. (Figure created in Corel Draw X4 (v.14.0.0.567) by Łucja Fostowicz-Frelik).

Principal Components 1 and 2 (PC1 and PC2) represent 87.48% and 5.75% of the variance, respectively, whereas Principal Components 3–4 represent further 4% of the variance (Supplementary Table S2). All the variables are positively correlated with PC1 and their loadings are very balanced (Fig. 4). Thus, it implies that the PC1 represents a proxy for the size of the bone. PC2 is most strongly correlated with the length of the calcaneal body, BL (-0.86) and more weakly correlated with the width of the cuboid facet (CCW) and anterior width of the ectal facet (AEW), 0.31 and 0.21, respectively (Fig. 4). The correlation with the length of the calcaneal body is an especially important factor for estimating an animal’s vertical jumping ability; the species with elongated calcaneal bodies are generally better jumpers (see^8,36^). The strong negative correlation of the length of the calcaneal body in the second component is illustrated by grouping the species with a strong jumping locomotor repertoire (e.g., squirrels and chinchillas) towards the left side of the plot (Fig. 4). Incidentally, this phenomenon does not concern the calcanei of ricochetal species (see the position of *Pedetes* versus that of *Sciurus* and *Chinchilla*: Fig. 4), where the mechanics of a jump are differently realized, and the stabilisation and relative stiffness of the ankle joint plays the most important role (thus, the calcaneal body and calcaneal tuber are more similar in size).

In the plot of PC1 against PC2, ischyromyids do not cluster together. Instead, the PCA morphospace is divided into two (or even three) broad groups of ischyromyid locomotor adaptations: the ambulatorial species and those with more pronounced jumping or cursorial ability. Chinese taxa fall among typically large ambulatorial rodents, such as the coypu (*Myocastor*) and porcupines (*Atherurus* and *Hystrix*). Closest to them there is the North American ischyromyid *Quadratomus*, which is somewhat shifted towards the cursorial species and can be thus distinguished as differently specialized (more cursorial). Two other North American ischyromyids, *Ischyromys* and *Reithroparamys*, are grouped with *Chinchilla* and *Ondatra*, respectively, which may imply some jumping and slightly scansorial locomotor adaptations for *Ischyromys* and those of typical agile generalist species for *Reithroparamys*.

Although the sample is limited, the results of the PCA analysis point to general differences in the structure of the calcaneus, and thus, locomotor specialisation, between Asian and North American ischyromyid species. Moreover, Asian species seem to differ less from each other than the North American ones do, reflecting the overall greater species diversity and coverage of a wider niche spectrum of the North American ischyromyids.

### Functional and paleoecological implications

The studied calcanei add to our knowledge on the functional aspects of locomotion of ischyromyid rodents. Proximal tarsal morphology has been recently used to interpret the locomotor behavior of some extinct rodents (see e.g.,^8,37–39^). In the scheme of locomotor categories of Samuels and Van Valkenburgh^40^, attributions proposed for early ischyromyids fit into generally terrestrial^41^, arboreal^42^ or a mixture of those two^35^.

A relatively short calcaneal body, widely spread sustentaculum tali, and a large peroneal process observed in most ischyromyid species (including these studied herein) indicate rather poor cursoriality. Instead, their ankle joint structure allows for a large freedom of foot movements in different planes. A medially extended sustentaculum tali together with a long and helically twisted ectal facet indicate a large degree of sliding between the calcaneus and astragalus along their articular facets, which makes possible a great degree of foot torsion resulting in foot eversion and inversion. This effect is further enhanced by an extended calcaneocuboid facet that is gently concave and oriented perpendicularly to the long axis of the calcaneus in species A.

Such adaptations are helpful for both clinging to branches and adjusting to uneven or inclined substrate during climbing. A great degree of freedom of movement may be helpful also during burrowing, when the hind legs are used to push forward loose soil out of a burrow or an animal is forced to maintain a crouched posture, when it digs with its forelegs and head. Nevertheless, as much as the calcaneal structure may suggest some burrowing ability in ischyromyids (see Rose and Chinnery^35^), the rest of the postcranial skeleton known from the more complete specimens of North American representatives^41^ does not support fossorial adaptations. In particular, a long tail in the pre-Oligocene North American (see e.g., *Paramys* or *Reithroparamys* in Wood^41^: figs. 8 and 44, respectively) suggests some arboreal adaptations or at least occasional climbing, as such a tail greatly enhances balancing on uneven terrain. In contrast, typically fossorial mammals have reduced tails^43^.

The overall morphology of dental and mandibular remains^16,18^ of Asian ischyromyids is similar to that of their North American counterparts^16,19^. As complete or even partial postcranial skeletons are unknown for the Asian ischyromyids, we can surmise their general locomotor adaptations based on calcaneal morphology which, although not in striking contrast with their North American counterparts, shows some differences.

Overall, the calcaneal morphology of Chinese ischyromyids is closest to that of ground squirrels and especially porcupines (both *Atherurus* and *Hystrix*) and the coypu; the similarity to the last one is supported also by the PCA analysis. The calcaneal morphology and proportions may therefore reflect their locomotion behavior as generalized terrestrials, with a somewhat limited ability to climb (a rare but observed behavior in *Hystrix*) and to dig burrows (as does *Atherurus*^43^). A transverse and gently concave calcaneocuboid facet of species A facilitates foot rotation along the long axis, useful on an uneven, rocky terrain or while traversing branches, when an animal needs a flexible foot for a better grip (see Chester et al.^44^). On the other hand, the lack of both a characteristically bent calcaneal tuber and posteriorly located peroneal process in all ischyromyids (except for *Notoparamys*, see Rose and Chinnery^35^) argues against the arboreal adaptations characteristic of tree squirrels.

## Discussion

In contrast to the preceding fauna (Arshantan ALMA) of Nei Mongol, where the Glires assemblages were less diverse and dominated by the early ctenodactyloid *Tamquammys*^8,10,19^, the early middle Eocene (Irdinmanhan ALMA) witnessed a surge of new Glires taxa including, among others, ischyromyids^29^. In particular, ctenodactyloids diversified markedly in that interval^10,29^ and the first cricetids appeared^45^, along with the increase in lagomorph diversity^46,47^ and a slight mimotonid revival^48,49^. Mimotonids were much less abundant during the Arshantan and Irdinmanhan than in the Bumbanian ^25^, although their taxonomical diversity was higher ^48,49^.

Since their origin, ischyromyids were relatively large, based on their first molar length, which reached frequently over 4 mm^6^. Thus, in the Irdinmanhan biocenoses their closest ecological competitors should be other large Glires, such as *Gomphos* and *Mimolagus*^48^, although by then *Gomphos* was already much less abundant than in the preceding Bumbanian ALMA^25,26,50^. Among the Irdinmanhan rodents most ctenodactyloids (apart from *Yuomys cavioides* and *Y*. *weijingensis*) and cricetids were small, based on the M1/m1 length much below 2 mm^19,45^. The lagomorphs were even smaller^47^; their body mass was estimated at less than 150 g ^48^. Thus, these herbivores probably were not direct competitors of ischyromyids.

**Figure 5:**
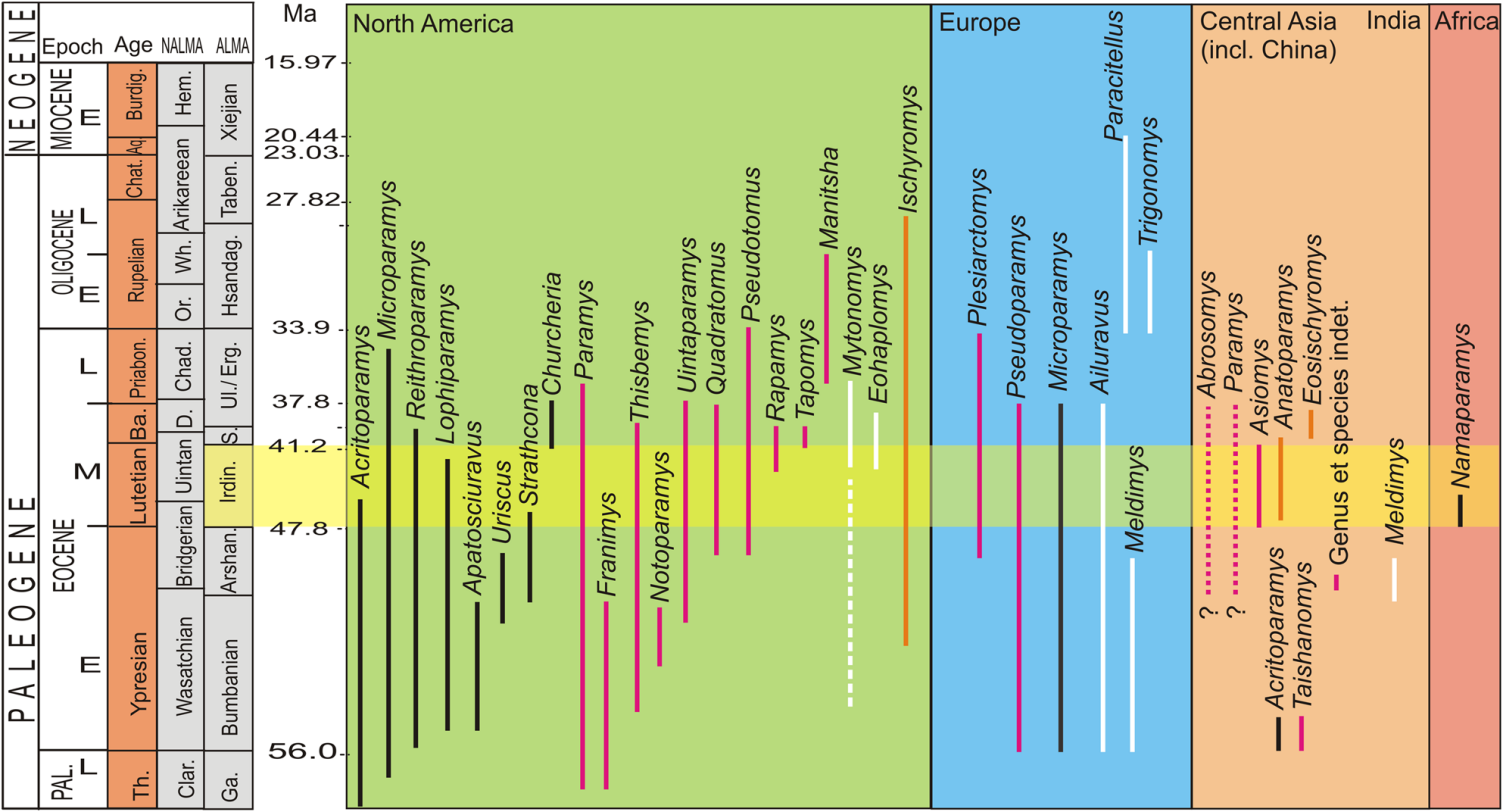
Temporal ranges of Ischyromyidae. Color of range line denotes subfamily: black, Microparamyinae; pink, Paramyinae; orange, Ischyromyinae; white, Ailuravinae; yellow stripe marks the Irdinmanhan ALMA. Geological and stratigraphic chart based on Woodburne^11^, Ni et al.^52^, and Cohen et al.^64^; classification based on Anderson^6^ and McKenna and Bell^65^. For fossils age data, see the text. (Drawing made in Corel Draw X4 (v.14.0.0.567) by Łucja Fostowicz-Frelik).

Asian ischyromyids are relatively rare compared to the North American record; in total, ten genera were reported from Asia (Fig. 5). Six taxa come from China^16,19,21,30,51^, one from India^22^, two plausible from Kazakhstan^20^, and one from Pakistan (see Dawson^17^:101). Thus far, all of them have been known either from isolated teeth or mandible fragments^16^. The first Chinese ischyromyids *Taishanomys changlensis* and *Acritoparamys*? *wutui* are known from the early Early Eocene of the Wutu Formation, Shandong Province, China^16,18^. The Wutu Fm. is coeval with the upper Nomogen Formation in Nei Mongol^52^, and characterized by the presence of Alagomyidae, an important faunal element typical of the Bumbanian and preceeding Gashatan ALMAs^2,25,53^. Early ingress of ischyromyids into the Erlian Basin in Nei Mongol (Nuhetingboerhe site) took place in the Arshantan^19^. The group, however, did not become established in this area until the beginning of the Irdinmanhan^19^. The northward expansion of ischyromyids during the middle Eocene to early Oligocene interval in China mimics the trend observed in other mammalian groups, in particular anagalids, a Paleogene group of primitive Euarchontoglires endemic to Asia^54^, which also appeared in Nei Mongol not earlier than in the Irdinmanhan^49,55^.

The affiliations of Chinese ischyromyids are vague, as well as are the exact routes of their dispersal. *Taishanomys* and *Acritoparamys* from the Wutu Formation, *Anatoparamys* from Shanghuang, and *Asiomys* from Nei Mongol are assigned to Paramyinae^16,19,30,33^. *Eoischyromys* from the middle Eocene of the vicinity of Beijing^21^ is considered Ischyromyinae. *Hulgana* from the Ulan Gochu Formation, Jhama Obo, Nei Mongol^56^, once considered an ischyromyine, may be a cylindrodont^57^. Furthermore, the lack of Ailuravinae in the Chinese^19^ and Central Asian fossil rodent record^20,58^ indicates that ischyromyids came to China with a different wave of migration than that which led to colonization of India^22^. The presence of Ischyromyinae and a greater similarity of *Asiomys* to North American ischyromyids than to European taxa^19^ imply that the migration route to northern China may have been directly from North America via the Beringian region, as for tarkadectine primates^59^.

The overall scarce ischyromyid findings in Asia indicate that the group did not adapt well to Asian environments and may have suffered from competition with small herbivores, either mimotonids or even minute perissodactyls, abundant in China at that time^48,60^. Also, it seems that early Asian ischyromyids preferred more humid and warm habitats; they were most diverse in the middle Eocene Shanghuang fissure fillings, southern China (five taxa;^30^), which yielded a rich mammalian fauna suggestive of a treed environment (e.g., bats and primates;^61^). The demise of ischyromyids in Asia may have been connected with a growing ecological pressure from other rodent groups, especially ctenodactyloids, which continued to dominate the late Eocene of China^17,62^.

## Material and methods

The anatomical terminology (Figs. 2, 3) follows Fostowicz-Frelik^36^, Ginot et al.^37^, and Fostowicz-Frelik et al.^8^. The measurements (Fig. 2, Table 1, Supplementary Information) were taken with a digital caliper (with 0.1 mm accuracy). The comparative material used in this paper includes postcranial material of ischyromyid rodents from the AMNH collection as well as extant rodent taxa (coll. AMNH, ISEZ, IVPP, and ZMCAS; see Supplementary Information) chosen to include the calcanei of large extant taxa representing ambulatorial, semi-fossorial, ricochetal, cursorial, and arboreal locomotor adaptations. PCA analyses on the variance–covariance matrix were performed with PAST v. 2.17^63^.

### Geological settings

The ankle bone material studied here comes from three localities (Fig. 1) of the Erenhot area in the Erlian Basin^28^, the classical area of the Central Asiatic Expeditions since 1923^27,28,60^. These are: Irdin Manha Escarpment, Daoteyin Obo (= Overnight Camp; see Wang et al.^28^: Fig. 1), and Huheboerhe (= ‘10 miles Southwest of Camp Margetts’). The stratigraphic range of these localities spans generally the upper Eocene (Bumbanian Asian Land Mammal Age [ALMA]) strata assigned to the Nomogen Formation through the beds of the lower middle Eocene belonging to the Irdinmanhan Formation^26–28^. The Huheboerhe outcrop covers the whole mentioned sequence, while the Irdin Manha Escarpment and Daoteyin Obo sections include only the Arshantan–Irdinmanhan beds, but the thickness of the Arshantan strata varies (Fig. 1). The material studied herein comes exclusively from the lower Irdinmanhan (IM-1) part of the section (Fig. 1). The specimens were collected, mostly through surface screening, by the IVPP field parties during 2004–2012 seasons. GPS data (geographic coordinates and the height above sea level) were recovered for each finding.

## Supplementary Information

Supplementary Information.

## Data Availability

All data generated or analysed during this study are included in this published article (and its Supplementary Information files).
