# Supplementary material for: Tarsal morphology of ischyromyid rodents from the middle Eocene of China gives an insight into the group’s diversity in Central Asia
Source: Sci Rep. 2021 Jun 2;11:11543. doi: 10.1038/s41598-021-90796-1 (PMC8172891; doi:10.1038/s41598-021-90796-1)

Supplementary Information

Tarsal morphology of ischyromyid rodents from the middle Eocene of China gives an insight into the group's diversity in Central Asia

Łucja Fostowicz-Frelik[1, 2, 3*], Sergi López-Torres[3, 4, 5], and Qian Li[1, 2]

[1] Key Laboratory of Vertebrate Evolution and Human Origins, Institute of Vertebrate Paleontology and Anthropology, Chinese Academy of Sciences, Beijing, 100044, China

[2] CAS Center for Excellence in Life and Paleoenvironment, Beijing, China

[3] Institute of Paleobiology, Polish Academy of Sciences, Warsaw, PL-00-818, Poland

[4] Division of Paleontology, American Museum of Natural History, New York, NY 10024, USA

[5] New York Consortium in Evolutionary Primatology, New York, NY, USA

Correspondence and requests for materials should be addressed to ŁF-F (lfost@twarda.pan.pl)

Supplementary Table S1. Measurements (in mm) of the comparative rodent taxa used for PCA analysis. For measurement abbreviations, see Fig. 2 in the main text

**Species**	**Specimen No.**	**CL**	**CW**	**BL**	**TW**	**5TT**	**EL**	**AEW**	**TEW**	**CMT**	**BW**	**CCW**	**CCL**	**TWM**	**TL**
Atherurus macrourus	MZCAS	24.20	11.60	6.40	7.50	9.10	8.00	4.90	5.80	10.10	7.80	5.30	7.40	6.60	13.40
Castor fiber	ISEZ M/13506	55,83	27,68	5,64	22,31	12,46	11,32	8,38	12,4	15,18	17,57	12,17	8,04	18,45	31,69
Cavia porcellus	ISEZ M/11855	11,5	5,57	3,65	3,72	2,81	3,77	1,43	2,68	3,23	3,34	2,17	2,36	3,68	6,01
Chinchilla lanigera	ISEZ M/11385	13,51	5,59	4,26	3,64	3,58	4,69	1,39	2,39	5,59	4,65	1,91	2,98	3,53	6,43
Chinchilla lanigera	ISEZ M/11387	13,59	5,31	4,47	3,5	3,84	5,09	1,44	2,61	5,47	4,36	2,03	3,34	3,14	6,52
Chinchilla lanigera	ISEZ M/13331	13,7	5,2	4,22	4,05	3,78	5,7	1,43	2,6	6,22	4,42	2,05	2,82	3,26	6,71
Chinchilla lanigera	ISEZ M/13647	12,71	5,48	4,6	3,81	3,71	5,05	1,79	2,59	5,46	4,63	2,72	3,3	3,72	5,76
Cricetus cricetus	ISEZ M/13323	10,45	5,68	1,52	2,98	3,73	3,36	1,48	2,04	3,33	4,45	2,64	2,76	2,2	6,42
Cricetus cricetus	ISEZ M/13426	8,20	5,04	0,98	2,23	2,83	3,29	1,17	1,99	2,79	3,78	2,55	2,54	1,72	4,54
Cricetus cricetus	ISEZ M/4660/70	8,67	4,98	1,25	2,05	2,58	3,41	1,56	1,71	2,6	3,52	2,23	2,28	1,67	4,8
Dasyprocta leporina	ISEZ M/11856	28,79	10,15	10,43	7,09	8,7	7,82	1,89	5,48	9,76	5,7	3,14	4,52	4,44	14,54
Dinomys branickii	AMNH M 70354	38,01	20,02	15,51	13,31	11,44	7,32	6,1	8,09	12,64	12,17	6,8	7,42	12,33	19,64
Dinomys branickii	AMNH M 85372	35,92	17,08	15,57	14,23	11,17	7,5	5,8	7,65	10,66	13,19	8,26	6,74	11,16	15,91

Hydrochoerus hydrochaeris	AMNH M 206452	71,43	28,62	22,19	21,28	23	19,33	7,76	14,1	22,94	19,6	15,05	12,9	13,9	32,62
Hydrochoerus hydrochaeris	AMNH M 206440	69,1	28,33	19,35	20,32	24,83	17,76	8,69	13,96	26,48	13,58	13,34	13,47	12,08	34,06
Hydrochoerus hydrochaeris	AMNH M 139225	46,32	22,35	11,47	13,2	16,59	15,56	6,66	11,34	17,96	13,3	8,83	7,56	9,79	22,27
Hydrochoerus hydrochaeris	AMNH M 23467	50,92	21,39	14,87	13,88	15,24	12,68	6,57	10,61	19,59	13,53	10,93	10,51	9,68	24,92
Hydrochoerus hydrochaeris	AMNH M 214638	66,93	25,96	18,07	17,58	23,59	16,99	7,84	13,18	26,92	12,22	10,66	12,52	11,36	35,24
Hydrochoerus hydrochaeris	AMNH M 214624	71,22	32,33	20,04	19	23,1	18,34	9,23	13,91	28,13	13,1	11,72	12,18	12,47	36,02
Hydrochoerus hydrochaeris	AMNH M 209121	76,4	31,69	18,59	20,26	25,13	21,99	10,51	18,23	30,69	16,66	14,86	16,18	14,02	40,57
Hydrochoerus hydrochaeris	AMNH M 209115	75,14	29,69	19,64	19,77	25,8	18,9	7,65	13,67	28,67	17,38	14,3	14,83	12,65	36,16
Hydrochoerus hydrochaeris	AMNH M 209113	54,5	24,7	17,38	15,68	19,21	13,34	7,34	12,21	21,91	14,68	10,47	10,63	11,15	25,39
Hydrochoerus hydrochaeris	AMNH M 209107	64,22	27	18,23	17,33	20,59	18,03	7,26	12,52	24,77	13,47	15,44	13,52	10,67	30,27
Hystrix africaeaustralis	AMNH M 216337	34,37	21,8	8,18	12,62	15,06	10,25	7,4	9,42	15,9	13,79	9,13	7,25	11,07	20,38
Hystrix cristata	AMNH M 119506	35,63	19,3	9,4	10,48	14,01	12,66	7,96	9,82	14,78	12,85	7,31	8,15	9,73	16,25
Hystrix cristata	AMNH M 87220	34,3	20,06	7,54	10,78	15,43	12,4	7,54	10,19	16,02	13,72	9,67	7,3	8,29	15,41
Hystrix cristata	AMNH M 87222	35,27	22,84	6,86	11,6	18,76	12,93	6,52	9,41	15,02	13,82	8,97	7,65	8,82	19,87
Hystrix cristata	AMNH M 51735	36,38	19,54	8,01	11,51	14,87	10,72	6,18	9,76	14,09	14,52	7,15	7,68	9,95	19,14
Hystrix indica	AMNH M 14144	35,48	20,56	8,43	9,97	16,44	12,02	6,56	8,23	16,92	13,99	7,94	8,27	8,28	17,06
Marmota marmota	ISEZ M/1142/59	21,35	11,55	2,94	6,48	8,23	8,6	3,14	8,07	9,88	6,47	4,8	5,17	4,32	12,17
Marmota marmota	ISEZ M/1143/59	21,09	10,91	2,63	6,73	7,89	9,6	2,77	7,35	9,48	6,01	4,75	5,06	4,24	12,10

Myocastor coypus	AMNH M 148797	30,51	12,86	9,61	8,81	10,04	6,5	6,04	6,66	10,61	10,31	6,09	7,02	5,62	15,63
Myocastor coypus	AMNH M 206461	31,96	16,23	10,24	8,72	10,62	7,74	5,94	6,54	12,4	11,05	5,7	6,79	6,61	16,06
Myocastor coypus	AMNH M 206456	30,72	15,31	10,15	8,92	10,76	7,82	5,88	7,72	12,27	10,54	5,57	6,79	7,17	15,67
Myocastor coypus	AMNH M 206454	28,69	12,99	9,59	8,46	9,43	6,71	5,27	7,18	11,03	10,06	6,14	6,58	5,98	13,67
Myocastor coypus	AMNH M 35626	28,31	13,85	9,38	9,51	10,39	8,09	5,44	6,65	11,7	9,99	5,77	6,51	6,88	12,76
Myocastor coypus	AMNH M 35622	25,83	13,83	7,37	8,58	10,23	8,42	5,58	7	10,62	9,44	5,2	6,66	7,79	12,47
Myocastor coypus	AMNH M 35625	28,47	16,47	9,68	8,81	10,77	8,61	6,23	7,34	11,09	10,49	6,46	6,84	7,75	14,47
Myocastor coypus	AMNH M 206455	29,65	14,61	9,98	9,92	9,54	6,64	4,52	7,7	10,91	9,53	5,75	6,7	6,41	13,89
Myocastor coypus	AMNH M 19541	28,77	14,83	8,77	9,06	10,12	7,73	6,13	7,31	11,66	10,33	5,82	6,43	7,4	14,03
Myocastor coypu	IVPP Ni com. coll.	28.30	13.90	7.80	9.20	9.60	9.10	6.10	8.00	12,39	9.93	6.65	6.15	8.00	15.80
Ondatra zibethicus	ISEZ M/10373/90	15,98	8,88	4,85	4	5,55	5,42	2,12	3,86	6,98	7,06	4,57	4,45	3,93	8,41
Ondatra zibethicus	ISEZ M/10377/90	16,07	8,69	5,45	3,75	6,06	5,51	2,97	3,62	6,73	6,47	3,24	4,1	3,77	8,33
Ondatra zibethicus	ISEZ M/10378/90	15,53	8,55	4,3	4,57	5,35	5,48	2,55	3,6	7,07	7,4	3,71	4,44	4,06	8,41
Ondatra zibethicus	ISEZ M/10379/90	16,57	8,96	5	3,92	5,31	5,69	2,42	3,73	6,28	6,47	2,61	3,3	3,32	8,29
Ondatra zibethicus	ISEZ M/11726	15,83	8,6	4,34	3,95	5,25	6,05	2,1	3,54	6,89	6,6	3,76	3,66	3,87	7,72
Ondatra zibethicus	ISEZ M/2897/65	15,58	8,6	5,53	4,62	5,56	5,42	2,71	3,53	7,16	6,18	3,51	3,91	3,2	8,56
Ondatra zibethicus	ISEZ M/4869/74	15,53	8,1	4,48	3,91	5,61	5,23	2,15	2,84	6,73	6,46	3,25	3,69	3,8	8,5
Ondatra zibethicus	ISEZ M/4870/74	15,37	8,74	3,99	3,83	4,89	4,89	2,61	3,41	6,2	6,63	3,3	3,77	3,48	8,69

Ondatra zibethicus	ISEZ M/5635/80	15,02	8,28	4,41	4,27	5,38	5,57	2,06	3,3	6,45	6,39	2,83	3,94	3,73	8.00
Ondatra zibethicus	IVPP Ni com. coll.	17.80	7.30	6.50	4.30	6.20	3.60	4.00	4.70	7.20	6.20	4.70	3.50	3.40	10.10
Pedetes capensis	ISEZ M/11853	36,39	9,42	17,1	6,98	9,52	7,91	3,35	7,11	11,42	6,08	3,88	5,68	4,73	17,38
Pedetes capensis	ISEZ M/11854	34,19	10,19	14,84	7,19	9,24	7,66	3,15	7,56	10,44	6,75	3,01	4,97	4,96	17,14
Sciurus vulgaris	ISEZ M/12040	11,08	6,61	1,8	2,46	3,99	5	1,83	4,04	4,83	3,07	2,91	2,64	1,93	5,98
Sciurus vulgaris	ISEZ M/12119	11,35	6,58	2,25	2,32	4,02	5,28	1,48	4,05	4,37	3,17	2,82	2,32	1,77	6,43
Sciurus vulgaris	ISEZ M/13235	10,34	6,37	1,69	2,41	3,78	5,37	1,72	3,81	4,82	2,63	2,38	2,34	1,89	5,69
Sciurus vulgaris	ISEZ M/13321	10,78	6,3	2,16	2,42	4,18	5,09	1,49	3,91	4,54	3,5	2,79	2,23	2,01	5,74
Sciurus vulgaris	ISEZ M/13322	10,73	6,36	2,21	2,44	3,64	5,44	1,52	3,79	5,2	3,51	2,8	2,4	1,83	5,63
Sciurus vulgaris	ISEZ M/13440	10,32	6	2,1	2,09	3,54	5,08	1,48	3,91	4,35	3,35	2,51	2,51	1,6	5,44
Sciurus vulgaris	ISEZ M/13451	11	6,16	1,91	2,37	4,06	5,1	1,51	3,64	4,39	3,14	2,69	2,47	1,77	6
Sciurus vulgaris	ISEZ M/13453	11,64	6,84	1,88	2,53	4,16	5,61	1,95	3,92	5,02	2,76	2,71	2,58	2,36	6,39

Supplementary Table S2. Eigenvalues for PCA analysis, all components

PC	Eigenvalue	% variance
1	0.726286	87.622
2	0.0478198	5.7692
3	0.0199759	2.41
4	0.0165451	1.9961
5	0.0063876	0.77063
6	0.00436129	0.52616
7	0.00251195	0.30305
8	0.00179316	0.21633
9	0.00148945	0.17969
10	0.000891087	0.1075
11	0.000436289	0.052636
12	0.000181587	0.021907
13	0.000116941	0.014108
14	8.95778E-05	0.010807

Supplementary Figure S1. Loadings for the first four components

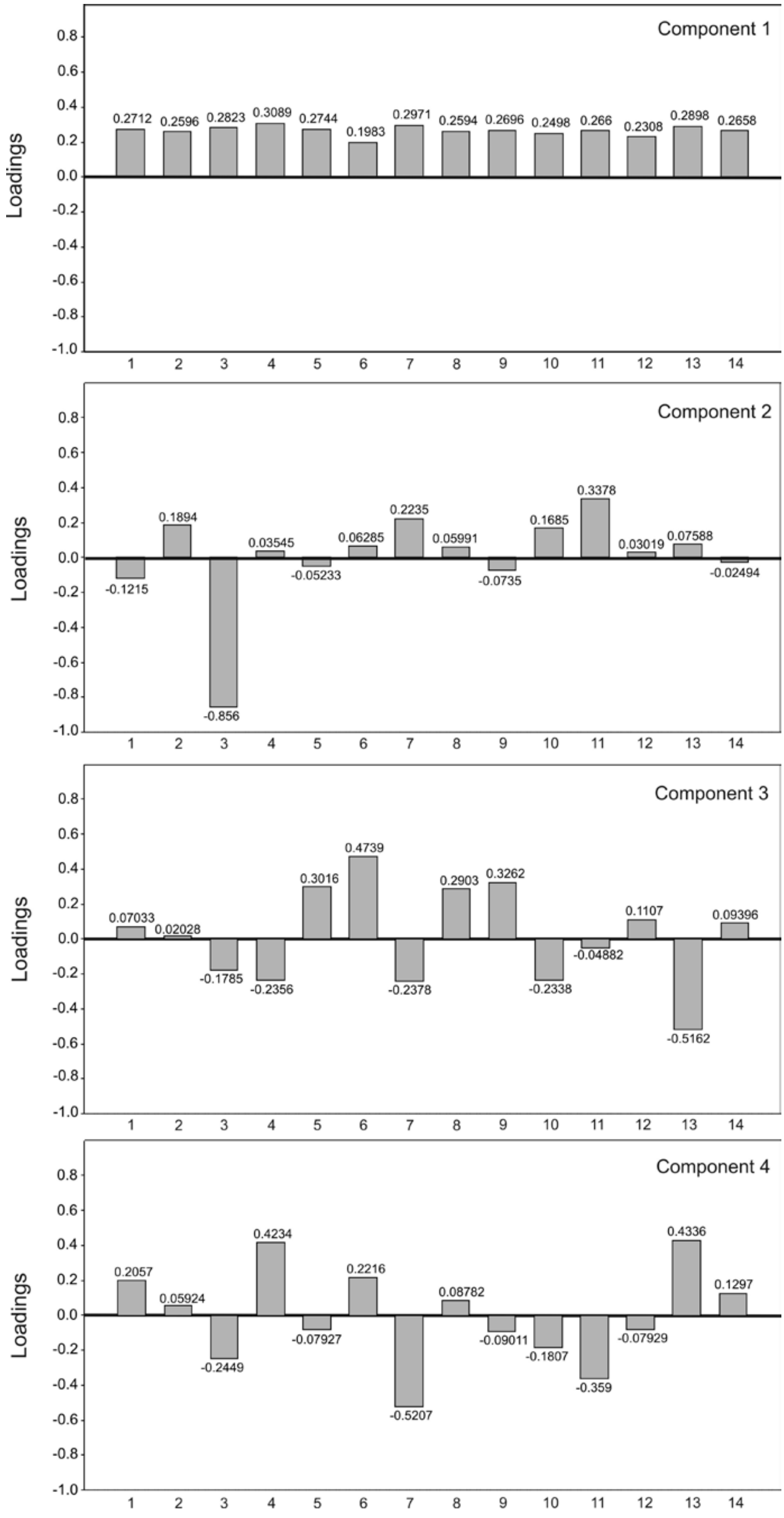

Supplement: Supplementary file 1 — Supplementary Information. [file 41598_2021_90796_MOESM1_ESM.pdf]
